# Fructose increases risk for kidney stones: potential role in metabolic syndrome and heat stress

**DOI:** 10.1186/s12882-018-1105-0

**Published:** 2018-11-08

**Authors:** Richard J. Johnson, Santos E. Perez-Pozo, Julian Lopez Lillo, Felix Grases, Jesse D. Schold, Masanari Kuwabara, Yuka Sato, Ana Andres Hernando, Gabriela Garcia, Thomas Jensen, Christopher Rivard, Laura G. Sanchez-Lozada, Carlos Roncal, Miguel A. Lanaspa

**Affiliations:** 1 Division of Renal Diseases and Hypertension, University of Colorado, Denver, CO USA; 2 Eastern Colorado Health Care System, Department of Veteran Affairs, Denver, CO USA; 3 Division of Nephrology, Clinica San Felipe, Lima, Peru; 4 Hospital Mateu Orfila, Menorca, Spain; 5 IUNICS-Idisba, University of Balearic Islands, Palma de Mallorca, Spain; 6 Department of Quantitative Health Sciences, Cleveland Clinic Foundation, Cleveland, OH USA; 7 Laboratory of Renal Physiopathology, Instituto Nacional de Cardiología, Ignacio Chávez, Mexico City, Mexico; 8 Division of Renal Diseases, University of Colorado Anschutz Medical Campus, Aurora, CO 80045 USA

**Keywords:** Fructose, Sugar, Sucrose, Kidney stone, Magnesium, Uric acid, Citrate, Oxalate

## Abstract

**Background:**

Fructose intake, mainly as table sugar or high fructose corn syrup, has increased in recent decades and is associated with increased risk for kidney stones. We hypothesized that fructose intake alters serum and urinary components involved in stone formation.

**Methods:**

We analyzed a previously published randomized controlled study that included 33 healthy male adults (40–65 years of age) who ingested 200 g of fructose (supplied in a 2-L volume of 10% fructose in water) daily for 2 weeks. Participants were evaluated at the Unit of Nephrology of the Mateo Orfila Hospital in Menorca. Changes in serum levels of magnesium, calcium, uric acid, phosphorus, vitamin D, and intact PTH levels were evaluated. Urine magnesium, calcium, uric acid, phosphorus, citrate, oxalate, sodium, potassium, as well as urinary pH, were measured.

**Results:**

Ingestion of fructose was associated with an increased serum level of uric acid (*p* < 0.001), a decrease in serum ionized calcium (*p* = 0.003) with a mild increase in PTH (*p* < 0.05) and a drop in urinary pH (*p* = 0.02), an increase in urine oxalate (*p* = 0.016) and decrease in urinary magnesium (*p* = 0.003).

**Conclusions:**

Fructose appears to increase urinary stone formation in part via effects on urate metabolism and urinary pH, and also via effects on oxalate. Fructose may be a contributing factor for the development of kidney stones in subjects with metabolic syndrome and those suffering from heat stress.

**Trial registration:**

ClinicalTrials.gov NCT00639756 March 20, 2008.

## Introduction

The lifetime risk of having a kidney stone has doubled in US adults during the last 40 years, and affects 8.8% of the US population [1, 2]. Two major reasons may account for the rise in kidney stones, with the first being the rise in obesity, metabolic syndrome, diabetes and hypertension, all of which increase the risk for kidney stones [3–8]. The second reason appears to be climate change [9]. Kidney stones are increased in hot environments [10] such as the southern United States (‘the stone belt’) [9]. While mean temperatures have increased by 0.8 to 1 °C in the last 75 years, the frequency of heat extremes has increased markedly [11, 12] and is coupled with increasing water shortages throughout the world [13]. Increasing heat places individuals who work outside at increased risk for heat stress and heat stroke [14, 15], as well as kidney stones and possibly chronic kidney disease (CKD) [9, 16, 17].

Studies on the urine of subjects with metabolic syndrome/obesity and subjects under heat stress, show similar risk patterns for nephrolithiasis. Subjects with metabolic syndrome have a urinary pattern that is characterized by low urinary pH, high serum uric acid, and normal or elevated urinary uric acid [18] which is similar to those exposed to heat stress [19], and both groups also show increased frequency of uric acid stones compared to the general population [19, 20]. Diabetic subjects also show a higher frequency of uricosuria and risk for uric acid kidney stones [8].

In searching for potential common risk factors for these two conditions, fructose jumps out as a possibility. Fructose is a simple sugar present in the two most common added sweeteners, sucrose (a fructose-glucose disaccharide) and high fructose corn syrup (HFCS), which is a mixture of fructose and glucose monosaccharides. Intake of fructose-containing sugars is epidemiologically and experimentally linked with the epidemics of obesity, metabolic syndrome and diabetes [21, 22]. Fructose is also generated in the kidney and hypothalamus under conditions of heat stress or dehydration due to the induction of aldose reductase, which converts glucose to sorbitol, followed by reaction with sorbitol dehydrogenase to generate fructose [23–25].

Epidemiological studies have linked dietary fructose [26] and sucrose [27] with an increased risk for kidney stones, despite the fact that natural fruits and some sodas are thought to be protective by providing citric acid (which generates citrate, an inhibitor of calcium-forming stones). Indeed, when one considers that fluid intake is a major strategy for reducing kidney stones [28], it is striking that the intake of sugary (fructose-containing) colas dose dependently increases the risk for stones, whereas intake of artificially sweetened colas shows an opposite relationship [29]. Indeed, the relationship between low fluid intake and kidney stones is present when the fluid intake is water, but not for soft drinks [29, 30]. Furthermore, a randomized trial reported a 10% reduction in stone recurrence for those who could reduce soft drink intake to < 24 oz/week [31].

While fructose intake increases the risk for kidney stones, the exact mechanism by which it does this is not known [32]. In this regard, a few years ago we performed a clinical trial in which healthy adult men were administered fructose with or without allopurinol for two weeks [33]. This allowed us to test the hypothesis that fructose might alter urinary constituents to favor calcium or uric acid stone formation.

## Methods

### Study design

The study was approved by the Ethics Committee of the Illes Balears Community (CEIC-IB; resolution: IP IB-850/07) and was registered on ClinicalTrials.gov [NCT00639756]. Participants were healthy nonsmoking males between the age of 40 and 65. Exclusion criteria included hypertension requiring the use of antihypertensive agents, diabetes mellitus, thyroid disease, any history of cardiovascular disease, gout, or cancer. In addition, any subject with an allopurinol allergy, a psychiatric disorder, who took lipid-lowering drugs, who was an alcoholic, who used illicit drugs, or who had a history of intolerance to fructose, was excluded. In order to reach a goal of 80 to 100 subjects, 293 individuals were interviewed, of whom 83 met the inclusion criteria and signed informed consent. Nine participants did not complete the study because of abdominal discomfort and diarrhea induced by fructose ingestion. Seven additional subjects were removed due to a history of kidney stones. Thirty-three were randomized (using a number system) to receive fructose (age 50.27 +/− 8.15, BMI 28.94 +/− 3.86), while the other thirty-four received fructose and allopurinol. The investigators were blinded at the time of allocation. As the two groups did not randomize evenly, we are presenting only the fructose alone group in this paper.

All subjects ingested 200 g of fructose daily, supplied in two bottles, each of 1 L capacity, containing 10% (*w*/*v*) fructose in water. Adherence to the study protocol was verified by collecting empty containers at each visit. During the study, subjects were asked to consume their usual diet, avoiding sugary drinks or alcohol.

### Sample preparation and analysis

A blood sample, a first-morning urine sample, and a 24-h urine sample were collected both before the study began and after the test concluded. The 24-h urine was collected according to the guidelines of the National Committee for Clinical Laboratory Standards (NCCLS); urine was preserved by addition of Thymol in isopropanol and refrigeration at 4 °C during the 24 h of collection. Blood and urine samples were processed immediately after collection.

### Laboratory analysis

Serum levels of glucose, uric acid, urea, creatinine, calcium, ionized calcium, magnesium, phosphorus, sodium, and potassium were measured in the Mateu Orfila Hospital laboratory using the Architect C-8000 Autoanalyzer from Abbott Diagnostics. Analysis of 24-h urine included measurement of urinary volume, urinary pH, and creatinine, urea, uric acid, phosphorus, sodium, potassium, oxalate and citrate. All analyses employed standard methods. To determine calcium and magnesium, urine samples were acidified with hydrochloric acid. Urine oxalic acid was measured at pH 3.8 (which captures both soluble and insoluble oxalate) whereas for urine uric acid, determination was done at pH 7.8 (which solubilizes any crystalline urate present). Urinary pH was measured using a micropH 2002 Crison pH-meter.

### Statistical analysis

Results are presented as means ± standard deviation. We utilized paired t-tests to determine the difference in study parameters from baseline to follow up. A type-I error probability of 0.05 was utilized as a threshold for statistical significance for each analysis. Analyses were conducted utilizing the SPSS Statistics software (IBM SPSS Statistics version 22 for Windows; IBM, New York, USA).

## Results

The baseline and followup serum and urine chemistry results of the 33 subjects who received fructose are shown in Table 1. Ten of these subjects had mild abdominal symptoms consisting of bloating, flatulence or limited episodes of diarrhea that were considered to be minor side effects. Fructose administration resulted in a rise in fasting serum uric acid levels (*p* < 0.001) and a slight but significant reduction in ionized calcium (*p* = 0.003) with a mild rise in intact parathyroid hormone (PTH) (*p* = 0.04). The primary effect of fructose on urinary parameters was to reduce urinary pH (*p* < 0.02), increase urine oxalate (*p* = 0.016), and reduce urine magnesium (*p* = 0.003). There was also an approximately 15% reduction in urine citrate and nearly 10% reduction in urinary phosphorus, as well as a 10% increase in urinary uric acid, but the differences did not reach significance.

**Table 1:** Characteristics of Subjects

	Baseline	Followup	Change	*P* value
Serum				
Calcium ionized (mmol/L)	1.248 ± 0.051	1.231 ± 0.047	−0.017 ± 0.030	0.003
25(OH) Vit D_3_ (ng/mL)	40.88 ± 17.80	37.03 ± 14.13	−3.85 ± 15.75	0.17
Intact PTH (pg/mL)	45.94 ± 18.09	52.42 ± 26.32	6.47 ± 17.58	0.042
Magnesium (mmol/L)	0.914 ± 0.073	0.909 ± 0.080	−0.005 ± 0.063	0.68
Phosphorous (mg/dL)	3.22 ± 0.47	3.28 ± 0.52	0.06 ± 0.38	0.35
Urate (mg/dL)	5.11 ± 1.37	6.13 ± 1.42	1.01 ± 0.75	< 0.001
Urine				
Urine Volume (mL/24 h)	1765 ± 659	2156 ± 737	391 ± 698	0.003
Urine pH	5.78 ± 0.76	5.52 ± 0.63	−0.26 ± 0.61	0.019
Calcium excretion (mg/24 h)	239 ± 129	214 ± 104	−25 ± 90	0.12
Magnesium excretion (mg/24 h)	92.1 ± 36.3	70.5 ± 35.6	− 21.6 ± 37	0.01
Phosphorus excretion (mg/24 h)	965 ± 349	878 ± 330	− 88 ± 319	0.12
Oxalate excretion (mg/24 h)	26.5 ± 18.1	37.6 ± 34.1	11.1 ± 25.0	0.016
Urate excretion (mg/24 h)	659 ± 269	724 ± 301	65 ± 246	0.14
Citrate excretion (mg/24 h)	760 ± 444	645 ± 311	−105 ± 462	0.16
Creatinine excretion (mg/24 h)	1700 ± 409	1766 ± 417	66 ± 317	0.24
Potassium excretion (mEq/24 h)	78.3 ± 31.5	71.6 ± 20.9	−6.7 ± 27.5	0.17
Sodium (mEq/24 h)	199.5 ± 77.4	187.8 ± 83.5	− 11.7 ± 81.9	0.42

## Discussion

Fructose and sucrose intake increase the risk for kidney stones [26, 27], but the mechanisms are not well understood [32]. Here we evaluated the effects of high doses of fructose (200 g/d, equivalent to a 6 pack of 20 oz colas) to mostly overweight adult males for two weeks. The primary effect of fructose on the plasma was an increase in serum uric acid, as previously noted [33]. In addition, there was a statistically significant but mild decrease in ionized calcium with an increase in PTH levels. The primary effect of fructose on the urine consisted of a significant reduction in urinary pH. In addition, there were statistically significant decreases in urinary sodium, potassium, and magnesium.

### Uric acid

Fructose generates uric acid during its metabolism, and serum urate [34–36] and urinary uric acid [37] increase acutely following fructose ingestion [34–36], especially during the postprandial period [38]. A high fructose intake is associated with higher serum uric acid levels and an increased risk for gout [39–41]. In our study there was a significant increase in serum uric acid, but while urinary uric acid was approximately 10% higher, it did not reach significance.

Subjects with metabolic syndrome and/or gout typically have a low fractional excretion of uric acid [42]. While urine uric acid levels are often in the normal range in these individuals, a purine load results in a rapid increase in uricosuria [43]. Likewise, fructose intake may also result in transient uricosuria related to acute rises in serum and urine uric acid following fructose metabolism [38]. Acute heat stress also causes transient rises in serum and urinary uric acid levels, in association with the development of urate crystalluria [19, 44, 45]. Thus, while a high urinary uric acid may not be commonly observed in subjects with metabolic syndrome and kidney stones, it may well be because 24 h measurements likely include periods of postprandial periods where urinary levels could be high varying with fasting periods where urinary urate levels fall back into the normal range.

### Low urine pH

Fructose ingestion was associated with a significant decrease in urine pH. Fructose metabolism is known to generate lactic acid, thereby providing an increase in acid load to the kidney [46]. However, another mechanism might be an impairment in ammoniagenesis, as has been shown to occur in metabolic syndrome [47, 48]. Ammoniagenesis occurs primarily in the proximal tubule, which is the major site where fructose is metabolized. Proximal tubular injury can be demonstrated in rats fed fructose [49] and also in mice that metabolize endogenously produced fructose from heat stress [23]. Hence it is possible that the low urine pH is the consequence of increase acid load (lactate) with an impaired ammoniagenesis response related to proximal tubular dysfunction.

### Serum calcium and PTH axis

We also found a small decrease in serum calcium, consistent with the observation that fructose can impair calcium absorption in the intestine [50]. This was associated with a small rise in PTH levels. Interestingly, we reported an association of serum uric acid with elevated PTH, and also found that uric acid can inhibit 1-alpha hydroxylase that converts 25 hydroxy vitamin D to 1,25 vitamin D [51]. In our study we did not measure 1,25 vitamin D; but the data, even though mild, would be consistent with an effect of fructose on this pathway. Nevertheless, the clinical significance of these findings can be questioned, as the changes in calcium and PTH were quite mild.

### Increased urinary oxalate

We also observed higher urinary oxalate excretion in subjects given fructose. The mechanism is likely a consequence of the increased glyoxylate synthesis that has been reported with increased fructose consumption [52]. Fructose has also been shown to increase oxalate synthesis in cultured rat hepatocytes [53]. In a study of seven healthy individuals, intravenous infusion of 35 g of fructose increased urinary oxalate excretion by 60% compared to glucose infusion [54]. On the other hand, these same authors found a decrease in urinary oxalate following oral fructose administration [55]. In our study, which used higher doses of oral fructose, and there was a clear increase in urinary oxalate excretion that would be consistent with its known ability to increase glyoxylate.

### Reduced urinary citrate

We also observed a 15% reduction in urinary citrate excretion which did not reach significance. Urinary citrate levels are mainly determined by the rate of proximal tubular citrate reabsorption and metabolism [56]. One key mechanism for hypocitraturia is increased intracellular citrate utilization by activation of adenosine triphosphate (ATP) citrate lyase (ACL) in the proximal tubule [57]. Both fructose and uric acid increase ACL activity in hepatocytes [58]. Since circulating fructose is rapidly filtered and reabsorbed via Glut5 and Glut 2 present in the proximal tubular brush border membrane [49], it is possible fructose could be increasing the synthesis of renal ACL activity directly. In addition, renal tubular ACL is activated by chronic metabolic acidosis and potassium deficiency [57], and hence the decrease in urinary pH and decrease in potassium excretion observed in our patients might also be increasing ACL via that mechanism.

### Other changes

Fructose intake was associated with a nonsignificant decrease in phosphate excretion. Fructose is known to induce intracellular phosphate depletion due to the rapid phosphorylation of fructose, and we did observe a fall in urinary phosphate which could represent movement of serum phosphate into the liver or other organs. Finally, there were mild decreases in urinary magnesium. Magnesium is known to decrease the risk of stone formation by reducing absorption of oxalate or by forming soluble complexes with oxalate in urine [59, 60]. A reduction in urinary magnesium could be consistent with a state of mild magnesium depletion, possibly related to the rapid decrease in ATP levels that occurs in the liver during fructose metabolism [61]. A decrease in urinary magnesium could also predispose to kidney stones.

### Limitations

A limitation is that the amount of fructose administered was high and may not reflect the effects of fructose for the average person. However, sugar intake averages 13 to 15% of energy intake in the general population and in some groups, such as adolescent males, may approach 25% or more. Second, fructose was administered as a 2 l fluid and hence we relied on 24 h urinary excretion as opposed to urinary concentration to examine effects. Another limitation is that the amount of fructose ingested could have affected intake of other foods that could potentially explain some of the findings. The response of fructose could also vary depending on whether the subject is predisposed to calcium oxalate or uric acid stones. Some findings could potentially be subject to results of multiple testing and will need to be confirmed in future studies. A final limitation is that all subjects were adult men and hence our data may not be generalized to women.

## Conclusions

The finding that fructose can directly increase the risk for kidney stones by altering pH and urinary oxalate and magnesium may help account for why soft drink consumption predisposes to kidney stones. In addition, dietary fructose is also known to increase serum and urine osmolarity and vasopressin release [25, 62–64]. Indeed, there is some evidence that fructose tends to shift water intracellularly (where it likely associates with glycogen) while maintaining a high serum osmolarity [64]. Thus, it provides a mechanism for maintaining a low urine output, which may increase the risk for both kidney stones and acute kidney injury from urate crystalluria [44, 45]. This likely accounts for why the urine output only increased by approximately 400 cc despite the large fluid intake associated with fructose. It is interesting that low urine volumes [30, 65], low urine pH [66, 67], high serum uric acid [68], soft drinks [69], and elevated serum osmolarity [70] all predict the development of CKD in addition to kidney stones, and that the epidemic of kidney stones and CKD appear linked [71, 72]. It is possible that the protective effects of bicarbonate in CKD are not due to neutralization of metabolic acidosis as much as it is the neutralization of the pH of the urine. We recommend further studies investigating how alterations in urinary constituents from fructose may influence both kidney stones and CKD.

## Abbreviations

ACL: ATP citrate lyase
ATP: adenosine triphosphate
BMI: body mass index
CKD: chronic kidney disease
HFCS: high fructose corn syrup
NCCLS: National Committee for Clinical Laboratory Standards
PTH: parathyroid hormone
